# Draft Genome Sequence of *Psychrobacter aquaticus* Strain CMS 56^T^, Isolated from a Cyanobacterial Mat Sample Collected from Water Bodies in the McMurdo Dry Valley Region of Antarctica

**DOI:** 10.1128/genomeA.00918-13

**Published:** 2013-11-07

**Authors:** Gundlapally Satyanarayana Reddy, Sreenivas Ara, Aditya Singh, Anil Kumar Pinnaka, S. Shivaji

**Affiliations:** CSIR—Centre for Cellular and Molecular Biology, Habsiguda, Hyderabad, Andhra Pradesh, Indiaa; CSIR—Institute of Microbial Technology, Sector 39A, Chandigarh, Indiab

## Abstract

We report the 3.2-Mb draft genome sequence of *Psychrobacter aquaticus* strain CMS 56^T^, isolated from a cyanobacterial mat sample collected from a water body in the McMurdo Dry Valley region of Antarctica.

## GENOME ANNOUNCEMENT

*Psychrobacter aquaticus* strain CMS 56^T^ is a Gram-negative, nonmotile, halotolerant, psychrophilic bacterium, isolated from a cyanobacterial mat sample collected from various water bodies in the McMurdo Dry Valley region of Antarctica (1). Based on the 16S rRNA gene sequence similarity, the closest neighbor of *Psychrobacter aquaticus* CMS 56^T^ is *Psychrobacter vallis* CMS 39^T^, isolated at the same time, with a similarity score of 99.59%, followed by other members of the genus *Psychrobacter*, with similarity scores ranging from 99.05 to 89.41%.

Whole-genome sequencing of *Psychrobacter aquaticus* CMS 56^T^ was performed using the Roche 454 (FLX Titanium) pyrosequencing platform, which yielded a raw sequence of 136,405,203 bp in 411,734 reads, which is 40× coverage of the genome. Assembly of the raw sequencing data was carried out using GS De Novo Assembler (v 2.8), which yielded a genome sequence of 3,216,409 bp in 38 contigs. All of the contigs were larger than 561 bp, with the longest contig being 535,134 bp long with a G+C mol% of 42.63%. Annotation of the assembled genome was performed using RAST (Rapid Annotation using Subsystem Technology) (2), tRNA was predicted by tRNAscan-SE (v. 1.23) (3), and rRNA genes were predicted by RNAmmer (v. 1.2) (4).

The *Psychrobacter aquaticus* CMS 56^T^ draft genome sequence has 2,814 predicted coding regions, including one 5S rRNA, one 23S rRNA, one 16S rRNA, and 43 tRNA genes. In the annotation there are 41 genes encoding resistance against antibiotics and toxic compounds, including 6 genes for arsenic resistance, 2 genes for mercuric resistance, 4 genes for fluoroquinolone resistance, 5 genes for cobalt-zinc-cadmium resistance, and 12 genes for copper homeostasis. There are 80 genes for membrane transport, including 88 Ton and Tol transport systems. Several stress response genes (97 genes) were also predicted in the annotation.

The functional comparison of the genome sequence, performed using the SEED framework for comparative genomics (http://www.theseed.org), revealed that *Psychrobacter arcticus* 273-4, *Psychrobacter cryohalolentis* K5, *Moraxella catarrhalis* RH4, and *Enhydrobacter aerosaccus* SK60, with similarity score values of 542, 521, 368, and 281, respectively, are the closest neighbors of *Psychrobacter aquaticus* CMS-56^T^. The whole-genome sequence of *Psychrobacter aquaticus* CMS-56^T^, isolated from Antarctica, will help in our understanding of the adaptation of psychrophiles to harsh conditions.

### Nucleotide sequence accession numbers.

The draft genome sequence of *Psychrobacter aquaticus* strain CMS 56^T^ has been deposited in DDBJ/EMBL/GenBank under the accession number AUSW00000000. The version described in this paper is version AUSW01000000.
